# Case report: Successful sequential therapy of crizotinb and entrectinib in *ROS1*-positive non-small-cell lung cancer with brain metastasis in later-settings

**DOI:** 10.1097/MD.0000000000036591

**Published:** 2023-12-22

**Authors:** Wen Dong, Jinke Zhuge, Pengli Yu, Kai Liu, Mingxing Yang, Hongkang Wang

**Affiliations:** a Department of Respiratory Medicine, Hainan Cancer Hospital, Hainan Province, China; b Geneplus Beijing, Beijing, China; c Department of Respiratory Medicine, Hainan General Hospital (Affiliated Hainan Hospital of Hainan Medical University), Hainan Province, China.

**Keywords:** crizotinb resistance, entrectinib, *EZR-ROS1*, NSCLC

## Abstract

**Rationale:**

Crizotinib has been approved in many countries for the treatment of patients with advanced *ROS1*-rearranged non-small cell lung cancers (NSCLC). Entrectinib is a *ROS1* inhibitor that has been designed to effectively penetrate and remain in the central nervous system (CNS) and has been recommended as first-line therapy. Few reports have precisely described sequential crizotinb followed by entrectinib in patients with *ROS1* fusion in later settings.

**Patient concerns:**

A 56-year-old man with a history of occasional smoking visited our hospital with cough, sputum, and shortness of breath.

**Diagnosis:**

He was diagnosed with right lung adenocarcinoma (T4N2M1a, stage IV) after image and histological examination, without *EGFR* or *ALK* fusion mutation.

**Interventions:**

He received three prior lines of therapies, including chemotherapy, nivolumab monotherapy, and paclitaxel plus anlotinib, with progression-free survival (PFS) of 5, 2, and 11.5 months, respectively. Then the patient began to have headaches and dizziness, and brain magnetic resonance imaging showed multiple brain metastases. Next-generation sequencing (NGS) of the biopsy from neck lymph node identified EZR-ROS1 (1.25% abundance). After 2 months of crizotinib (250 mg daily) plus bevacizumab, all pulmonary and brain lesions decreased, but a small liver lesion was discovered. As treatment went on for another 4 months, the liver lesion continued to grow while other lesions kept decreased or stable state. NGS analysis on the peripheral blood found the disappearance of *EZR-ROS1* fusion and a new *NTRK2* mutation (c.5C>T, p.Ser2Leu, 0.34% abundance) without other targetable molecular alteration. He received entrectinib (600 mg daily) plus bevacizumab and achieved a partial response. After 7 months of therapy, examination revealed progression of brain lesions.

**Outcomes:**

The patient had a total PFS of 13 months from sequential crizotinib and entrectinib therapy.

**Lessons:**

A *ROS1*-rearranged NSCLC with CNS metastases responded to sequential tyrosine kinase inhibitors treatment of crizotinb followed by entrectinib. This report has potential implications in guiding decisions for the treatment after crizotinib resistance.

## 1. Introduction

Chromosomal rearrangements of *ROS1*, can lead to the formation of constitutively active fusion proteins which act as oncogenic drivers.^[1,2]^ *ROS1* fusions are identified in approximately 1 to 2% of non-small cell lung cancers (NSCLC) and define a distinct molecular subset of NSCLC.^[2,3]^ *CD74-ROS1* is the most common fusion variant detected among patients.^[4]^ Crizotinib has been approved for use in *ROS1*-positive NSCLC based on the PROFILE 1001 study, which observed a median progression-free survival (PFS) of 19.2 months.^[5,6]^ Resistance to crizotinib can be mediated by secondary mutations within the *ROS1* kinase domain, or by activation of alternative signaling pathways.^[7]^ An updated integrated analysis of 3 clinical trials of another ROS1 tyrosine kinase inhibitor (TKI), entrectinib, demonstrated a high level of clinical benefit and intracranial efficacy in *ROS1*-rearranged NSCLC.^[8]^ Considering the high risk of CNS metastases in NSCLC, entrectinib is recommended as a first-choice TKI rather than after progression on another TKI. Lorlatinib showed clinical activity in those previously treated with crizotinib and represented an important next-line targeted agent.^[9]^ Through the previous literature and case reports, sequential entrectinib after crizotinib in *ROS1*-positive NSCLC was rarely reported. Our case has provided valuable clinical evidence for sequential TKI therapy of crizotinb followed by entrectinib in patients with *ROS1* fusion.

## 2. Case report

**Figure 1.**
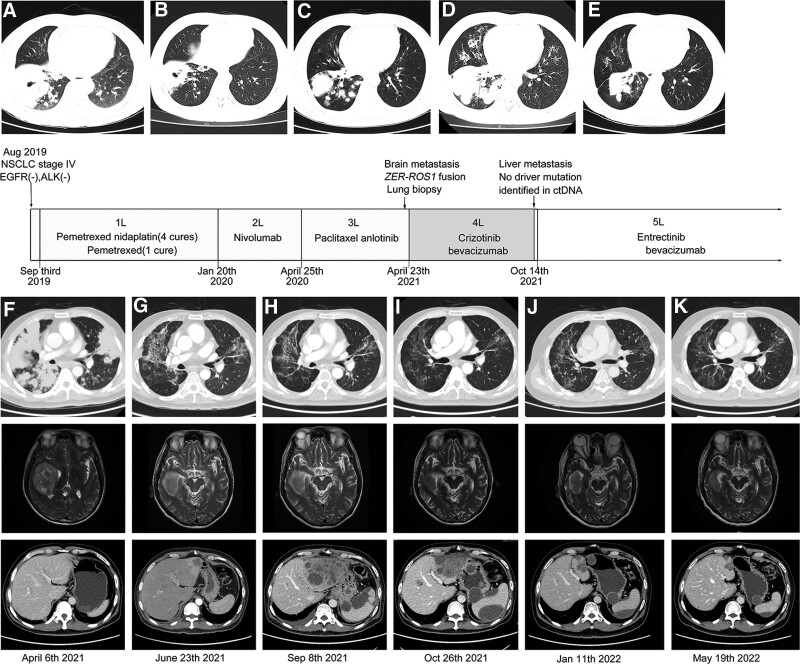
Timeline summary with dynamic imaging of the different therapeutic lines between August 2019 and May 2022. Baseline chest CT showing (A) a lung mass in the right lower lobe. Best response under chemotherapy with a partial response (B). Chest CT before second-line nivolumab beginning (C) and at disease progression (D). A partial response at 4 months after third-line therapy (E). Baseline chest CT and brain MRI (F) before crizotinib. Assessment showed a partial response of all lung lesions and brain lesions after 2 months of crizotinib (G), but new liver lesions (G, H). Chest CT and brain MRI before entrectinib beginning (I) and follow-up scans with partial response (J) and at brain lesions progression (K). CT, computed tomography, MRI, magnetic resonance imaging.

**Figure 2.**
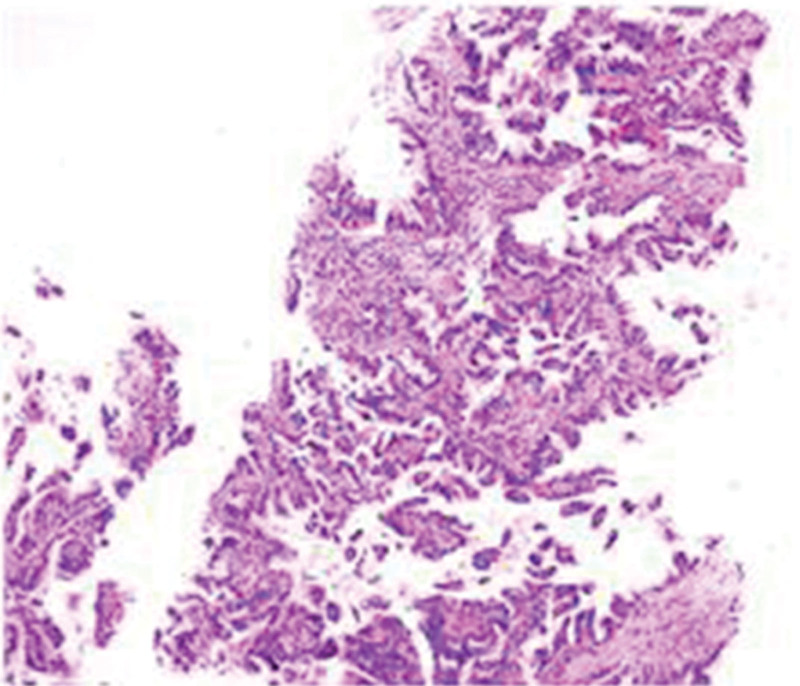
Pathological examination of the patient. Lung tissue biopsy specimen at diagnosis (Hematoxylin-eosin staining, magnification x10).

In August 2019, a 56-year-old male patient, with occasional smoking history, was referred to the Hainan Cancer Hospital with symptoms of cough and sputum which had persisted for 2 weeks, and shortness of breath that had persisted for 4 days. The contrast-enhanced computed tomography (CT) scan showed a 8.0 cm × 6.9 cm mass in the lower lobe of right lung, with multiple bilateral pulmonary nodules, hilum of right lung and mediastinal lymph node metastasis, and right sided pleural effusion (Fig. 1A). A right lung biopsy was obtained and established the pathologic diagnosis of invasive lung adenocarcinoma (Fig. 2). *EGFR* mutation and *ALK* tests showed negative results. Programmed death ligand 1 (PD-L1) expression was also negative after immunohistochemical (IHC) assay. Finally, his disease was diagnosed as right lung adenocarcinoma (T4N2M1a, stage Ⅳ), with an Eastern Cooperative Oncology Group performance status of 1.

The patient started to received first-line pemetrexed (850mg) plus nidaplatin (140 mg) after diagnosis. After 3 months, CT showed a partial response of the lung (Fig. 1B). He received 6 cycles of platinum-based doublets and 1 cycle of pemetrexed disodium maintenance therapy and experienced disease progression (PD, Fig. 1C), with a progression-free survival (PFS) of 5 months. Second and third-line therapies were prescribed: nivolumab (200 mg, PFS, 2 months, Fig. 1D) and then nanoparticle albumin bound paclitaxel (450mg) plus anlotinib (12mg daily, PFS, 11.5 months, Fig. 1E).

In April 2021, he began to have headaches and dizziness. The brain magnetic resonance imaging showed multiple brain metastases (Fig. 1F). At this time, a biopsy from neck lymph node was performed and showed metastasis from lung cancer. Next-generation sequencing (NGS) of the biopsy identified *EZR-ROS1*(E9: R33) (1.25% abundance). IHC showed positive expression of PD-L1, with tumor cell proportion score equal to 20% (SP263 antibody). On April 23,2021, the patient received crizotinib (250 mg daily). Assessment by CT at 2 months showed a decrease in all pulmonary and brain lesions, but a new liver lesion (Fig. 1G). Additional bevacizumab was added while continuing crizotinib. After 4 months of crizotinib, the liver lesion continued to grow while other lesions kept decreased or stable state. On 8 September, a new CT reassessment showed new and enlarged hepatic and splenic lesions, corresponding to a clear progression (Fig. 1H). Molecular analysis on the peripheral blood by NGS of DNA found the disappearance of *EZR-ROS1* and a *NTRK2* (c.5C > T, p.Ser2Leu, 0.34% abundance) without other targetable molecular alteration. Hence, after a molecular tumor board discussion, he received entrectinib (600 mg daily) plus bevacizumab on 14 October. Early assessment by CT at 12 days and at subsequent visits showed a partial response, with a decrease of all lesions, including hepatic and splenic lesions (Fig. 1I–K). The patient achieved a PFS about 7 months until progression of brain lesions (Fig. 1K). The patient had a total PFS of 13 months from sequential crizotinib and entrectinib therapy. Liquid biopsy of plasma blood revealed no driver mutations. Then the patient received additional radiation therapy for brain metastases and died of the end of November 2022 with overall survival of 38 months.

## 3. Discussion

*ROS1* is among the molecular biomarkers which should be analyzed at diagnosis in non-small cell lung cancer (NSCLC). Testing for these biomarkers is important for identification of potentially efficacious targeted therapies, as well as avoidance of therapies unlikely to provide clinical benefit. Tumor biopsy of the present case at diagnosis was only tested for *EGFR, ALK* and PD-L1, resulted in negative driver gene mutation.

The overall prevalence rate of *ROS1* fusion was 2.1% according to a cohort study of Chinese lung cancer patients.^[4]^ Advanced or locally advanced *ROS1*-rearranged NSCLCs achieved significantly longer median PFS (18.0 months vs 7.0 months, *P* < .001) from first-line crizotinib compared with first-line therapy chemotherapy.^[4]^ Another study in East Asian patients with *ROS1*-positive advanced NSCLC showed that crizotinib was beneficial in both first- and later-line settings.^[10]^ The presence of brain metastasis, other concomitant mutations in various genes, and single *CD74-ROS1* fusion might impair the crizotinib efficacy.^[4,10]^ The present case had uncommon single *EZR-ROS1* fusion, with no co-occurring driver mutations or tumor suppressor genes detected. *ROS1* resistance mutations were identified in 38% cases in the crizotinib-resistant biopsies, over one half of patients had unknown mechanisms of resistance to crizotinib based on gene sequencing alone.^[7]^ Several driver gene mutations were reported after crizotinib resistance in *ROS1* positive NSCLC. Our case detected no *ROS1* resistance mutation or driver gene mutation on liquid biopsy after progression on crizotinib. The discrepancies were identified between matched tumor and plasma analyses in certain cases, likely owing to multiple factors including tumor heterogeneity.^[7,11]^ The patient experienced liver progression while other lesions kept response. NGS of new biopsy on liver lesions might help reveal the mechanism of resistance.

Entrectinib is a multikinase inhibitor with antitumor activity against ROS1, ALK and pan-tropomyosin receptor kinase.^[12,13]^ Entrectinib demonstrated a high level of clinical benefit for patients with *ROS1* rearrangement NSCLC, including those with CNS metastases.^[8,14]^ National Comprehensive Cancer Network guidelines recommend ceritinib or crizotinib or entrectinib as preferred first-line tyrosine kinase inhibitors and lorlatinib or entrectinib upon progression.^[15]^ In patients with *ROS1* positive, around one third responded to lorlatinib after crizotinib resistance, with median PFS of 8.5 months.^[9]^ However, in patients previously treated with crizotinib, entrectinib is ineffective against the most frequently reported crizotinib-resistance mutation *ROS1* G2032R.^[16,17]^ Mutations other than *ROS1* G2032R might be responsible for crizotinib resistance. Few clinical cases described entrectinib treatment after cizotinib resistance. At present, the patient kept responding to entrectinib with a PFS of 7 months after resistance to fourth-line crizotinib therapy, which lasted for about 6 months. These results indicate that entrectinib may be useful after resistance to crizotinib with unknown resistance mechanisms through molecular analysis.

Although our result is promising, there are some limitations to this report. First, this report describes only 1 successful case; the underlined mechanisms of crizotinib resistance and entrectinib responsive to liver lesions remain unclear; whether other patients are sensitive to this sequential target regimen is still unknown. Further larger studies may be needed to confirm clinical activity of entrectinib in crizotinib-treated patients.

As a conclusion, this case emphasizes the potential benefit of sequential TKI therapy of crizotinb followed by entrectinib despite the absence of resistance mechanism in patient with *ZER-ROS1* fusion. However, as this report describes only 1 case, studies on more patients are needed to verify its effectiveness in future.

## Author contributions

**Data curation:** Jinke Zhuge, Kai Liu, Mingxing Yang, Hongkang Wang.

**Investigation:** Jinke Zhuge, Pengli Yu, Hongkang Wang.

**Supervision:** Wen Dong.

**Writing – original draft:** Wen Dong, Pengli Yu, Mingxing Yang.

**Writing – review & editing:** Wen Dong, Jinke Zhuge.
